# Using Patient-Reported Outcomes in Dose-Finding Oncology Trials: Surveys of Key Stakeholders and the National Cancer Research Institute Consumer Forum

**DOI:** 10.1093/oncolo/oyac117

**Published:** 2022-06-28

**Authors:** Julia Lai-Kwon, Alyssa M Vanderbeek, Anna Minchom, Olalekan Lee Aiyegbusi, Della Ogunleye, Richard Stephens, Melanie Calvert, Christina Yap

**Affiliations:** Drug Development Unit, The Institute of Cancer Research and Royal Marsden Hospital, London, UK; Clinical Trials and Statistics Unit, The Institute of Cancer Research, Sutton, UK; Drug Development Unit, The Institute of Cancer Research and Royal Marsden Hospital, London, UK; Centre for Patient-Reported Outcome Research, Institute of Applied Health Research, University of Birmingham, UK; National Cancer Research Institute Consumer Forum, UK; National Cancer Research Institute Consumer Forum, UK; Centre for Patient-Reported Outcome Research, Institute of Applied Health Research, University of Birmingham, UK; Birmingham Health Partners Centre for Regulatory Science and Innovation, University of Birmingham, UK; National Institute for Health Research (NIHR) Birmingham Biomedical Research Centre, NIHR Surgical Reconstruction and Microbiology Research Centre and NIHR Applied Research Collaborative West Midlands, University of Birmingham, Birmingham, UK; Clinical Trials and Statistics Unit, The Institute of Cancer Research, Sutton, UK

**Keywords:** drug development, cancer, quality of life, patient-reported outcomes, clinical trials, adverse events

## Abstract

**Background:**

Patient-reported adverse events may be a useful adjunct for assessing a drug’s tolerability in dose-finding oncology trials (DFOT). We conducted surveys of international stakeholders and the National Cancer Research Institute (NCRI) Consumer Forum to understand attitudes about patient-reported outcome (PRO) use in DFOT.

**Methods:**

A 35-question survey of clinicians, trial managers, statisticians, funders, and regulators of DFOT was distributed via professional bodies examining experience using PROs, benefits/barriers, and their potential role in defining tolerable doses. An 8-question survey of the NCRI Consumer Forum explored similar themes.

**Results:**

*International survey*: 112 responses from 15 September–30 November 2020; 103 trialists [48 clinicians (42.9%), 38 statisticians (34.0%), 17 trial managers (15.2%)], 7 regulators (6.3%), 2 funders (1.8%)]. Most trialists had no experience designing (73, 70.9%), conducting (52, 50.5%), or reporting (88, 85.4%) PROs in DFOT. Most agreed that PROs could identify new toxicities (75, 67.0%) and provide data on the frequency (86, 76.8%) and duration (81, 72.3%) of toxicities. The top 3 barriers were lack of guidance regarding PRO selection (73/103, 70.9%), missing PRO data (71/103, 68.9%), and overburdening staff (68/103, 66.0%). *NCRI survey*: 57 responses on 21 March 2021. A total of 28 (49.1%) were willing to spend *<*15 min/day completing PROs. Most (55, 96.5%) preferred to complete PROs online. 61 (54.5%) trialists and 57 (100%) consumers agreed that patient-reported adverse events should be used to inform dose-escalation decisions.

**Conclusion:**

Stakeholders reported minimal experience using PROs in DFOT but broadly supported their use. Guidelines are needed to standardize PRO selection, analysis, and reporting in DFOT.

## Teaser

There are limitations to clinician-assessed adverse events in early phase clinical trials, including under-reporting of lower grade toxicities. This article provides critical insights into the perspectives of clinicians, statisticians, trial managers, funders, regulators and consumers on the use of patient-reported outcomes in dose-finding oncology trials.

**Implications for Practice**

This study provides critical insights into the views of clinicians, statisticians, trial managers, funders, regulators and consumers on the inclusion of PROs to inform tolerable doses and regimens in DFOT. It has the potential to drive change in early phase clinical trial methodology by highlighting the value of PROs in providing the patient’s perspective on the type, frequency, and severity of adverse events. It also highlights that PRO data collected during DFOT can inform PRO objectives in later phase clinical trials, including efficient use of PROMs and sample size estimation. It identifies key questions for future research, including identifying the optimal setting in which to utilize PRO data, and calls for guidelines to standardize the use and reporting of PRO data to ensure confidence in the quality of PRO data and minimization of research waste.

## Background

Early phase (phase I and phase I/II) dose finding oncology trials (DFOT) establish the safety and tolerability of novel anti-cancer agents. Currently, adverse events are reported and graded by clinicians using the Common Terminology Criteria for Adverse Events (CTCAE). These data, in combination with dose modifications, discontinuations, and hospitalizations, are used to select tolerable doses and regimens to take forward into later phase studies. These include the maximum tolerated dose (MTD) and recommended phase II dose (RP2D).

It is well-recognized that there are limitations to clinician-assessed adverse events, including under-reporting of lower grade toxicities that are difficult to observe.^1,2^ This may result in an underestimation of treatment toxicities. Furthermore, therapeutic advances in immunotherapy and targeted therapy have produced challenges in defining tolerable doses. Such therapies may not produce toxicities in a dose-dependent manner and may result in toxicities that are longer in duration or occur later than the traditional dose-limiting toxicity (DLT) period.^3^ Low-grade toxicities experienced over the medium to long term may reduce a drug’s tolerability but are not taken into consideration when defining tolerable doses and regimens. Therefore, there is a clear need for alternative methods of determining tolerable doses and regimens that take the patient’s perspective on adverse events into account and can measure toxicities beyond the traditional DLT period.

Patient-reported outcomes (PROs) are measures of a patient’s health status that come directly from the patient.^4^ PRO measures may include generic or disease-related modules, but more recently, can also include single items from item libraries^5-7^ or single items assessing the overall impact of side effects on quality of life.^8^ PROs have been extensively studied in the clinical trial and routine care settings and are reliable, feasible, and valued by clinicians and patients.^9,10^ There is good evidence that they enhance the effectiveness of communication during the patient-clinician encounter and improve the detection of physical symptoms.^11^ This is highly relevant to DFOT, where accurately measuring adverse events is critical to determining the safety and tolerability of novel anti-cancer agents. In oncology clinical trials, PROs are increasingly evaluated to provide information about treatment risks, benefits, and tolerability.^12^ Patient-reported adverse events may be a useful adjunct to clinician-assessed adverse events for assessing a drug’s tolerability, providing complementary data to support the selection of tolerable doses and regimens.^13^

There is now increasing interest from clinicians, industry, and regulatory bodies to incorporate the patient’s experience in drug development through the use of PROs.^12,14,15^ A recent white paper by academics, industry, and regulatory experts recommended that the definition of drug tolerability be expanded to include direct reports from the patient about how they feel and function.^16^ The FDA issued guidance on the use of PROs in drug development and collaborated with the industry to form the PRO consortium with the aim of developing robust patient-reported symptom measurement tools.^4^ The FDA has promoted the use of the PRO version of the CTCAE (PRO-CTCAE) in cancer trials by creating an online repository for data visualization and ongoing work to standardize reporting of PRO-CTCAE on drug labels.^17^

There is limited literature regarding the current use of PROs in DFOT. A systematic review from 2012 to 2016 identified 15 phase I oncology trials with a health-related quality of life (HRQOL) endpoint.^18^ None of these studies used PRO data to inform the RP2D. We conducted a review of DFOT registered on ClinicalTrials.gov between 2007 and 2020.^19^ PRO endpoints were identified in more studies (an increase of 2.3 studies/ year, 95% CI, 1.6-2.9) from an increasing variety of countries over time. However, overall use remained limited, with only 29 studies in 2019 including a PRO endpoint.

Little is known about the reasons for limited PRO use in DFOT. In a qualitative study from the United Kingdom, patients and clinicians agreed that PROs could provide a more comprehensive view of a drug’s toxicity profile.^20^ However, clinical staff expressed concerns including monitoring PRO data, managing PRO data entry, and ensuring symptoms reported by patients were correctly attributed.

To explore these issues further, we surveyed international clinical trialists (clinicians, statisticians, trial managers/administrators), funders and regulators, and the National Cancer Research Institute (NCRI) Consumer Forum in the United Kingdom to evaluate attitudes toward the inclusion of PROs in DFOT.

## Methods

### International Stakeholder Survey

This was a cross-sectional, single time point, global online survey of international clinical trialists (clinicians, statisticians and trial managers/administrators) involved in the design, conduct, and/or analysis of DFOT working in hospitals, academia, or industry, as well as representatives from regulators or funders of DFOT. Participants needed to be able to complete an online survey without assistance.

#### Survey Content

The 35-question survey focused on prior experience in designing, conducting, and reporting DFOT involving a PRO endpoint, attitudes toward potential benefits and barriers to PRO use, and attitudes toward using PROs to define tolerable doses (Supplementary Text). Questions were informed by prior work examining trends in PRO use in DFOT^19^ and other studies examining PRO use in DFOT.^20,21^ The survey was hosted by a commercial survey tool provider (JISC Online Surveys) and used adaptive questioning (Supplementary Fig. S1). The survey was tested for usability by the Institute of Cancer Research staff prior to deployment. The Checklist for Reporting Results of Internet E-Surveys (CHERRIES) was used as a guide for survey design, distribution, and reporting (Supplementary Table S1).^22^

#### Survey Distribution

Ethics approval was received from the Royal Marsden Hospital Committee for Clinical Research. The survey was distributed via professional bodies, regulators, funders, and personal contacts (Supplementary Table S1). Initial contact with potential participants was via email. A generic link to the survey was provided. Eligibility was self-assessed based on the information provided in the introductory email and the first page of the online survey. Participation was optional and informed consent was inferred by submitting the survey.

#### Data Storage and Handling

No identifiable data was collected. Responses were only accessible to the study investigators via the password-protected JISC online platform.

#### Statistical Analysis

The survey was exploratory and aimed to survey at least 50 participants. Results were downloaded as comma-separated value files and analyzed by AV/CY using R (version 3.6.0). Descriptive statistics were used to summarise responses. The term “trialist” was used to denote a group including clinicians, statisticians, and trial managers. The term “participant” included all survey participants. Responses were analyzed for variation by work role, country of origin, and number of years of experience.

#### Free Text Analysis

Free text responses were analyzed by AV/ OLA using thematic analysis with a framework approach.^23^ This involved initial coding of 9 responses from 4 participants, comparison of codes and creation of a working analytic framework which was applied to the remaining data. Preliminary thematic analysis was discussed with all authors prior to establishing the final framework. All responses were included in the analysis.

### Survey of the NCRI Consumer Forum

This was a cross-sectional, single time point survey of NCRI Consumer Forum members (which include patients and carers who are affected by cancer and are involved with research activities) able to complete the survey via Zoom online polling. The NCRI Consumer Forum is a partnership between research funders, patients, and carers in the United Kingdom which promotes consumer involvement in cancer research. Consumers did not need to have participated in a DFOT to participate in the survey. Participants were shown a 20-min educational presentation on the use of PROs in DFOT prior to completing the survey.

#### Survey Content

The 8-question survey focused on prior experience participating in a clinical trial that collected PRO data, preferences regarding PRO collection (time willing to spend completing PROs, preferred method of PRO collection eg. paper, electronic), and attitudes toward using PROs to define tolerable doses.

#### Statistical Analysis

Results were analyzed by AV/CY using R (version 3.6.0). Descriptive statistics were used to summaries responses.

## Results

### International stakeholder survey

A total of 112 responses were received between 15 September 2020 and 30 November 2020. 103 trialists (48 clinicians (42.9%), 38 statisticians (34.0%), 17 trial managers/administrators (15.2%)], 7 regulators (6.3%), and 2 funders (1.8%) participated. In total 66 (58.9%) had worked in DFOT for 6–20 years, 33 (29.5%) for 0–5 years, and 13 (11.6%) for >20 years. Most worked in the United Kingdom (65, 58.0%) or United States (22, 19.6%) (Table 1).

**Table 1.** Global survey participant characteristics.

	Clinician (*n* = 48)	Statistician (*n* = 38)	Trial manager (*n* = 17)	Funder/regulator (*n* = 9)	Total (*n* = 112)
Years of experience					
0-2 years	7 (14.5%)	4 (10.5%)	3 (17.6%)	–	14 (12.5%)
3-5 years	3 (6.2%)	7 (18.4%)	8 (47.1%)	1 (11.1%)	19 (16.9%)
6-10 years	15 (31.2%)	12 (31.5%)	4 (23.5%)	4 (44.4%)	35 (31.2%)
11-20 years	16 (33.3%)	10 (26.3%)	1 (5.8%)	4 (44.4%)	31 (27.6%)
20+ years	7 (14.5%)	5 (13.2%)	1 (5.8%)	–	13 (11.6%)
Countries					
United Kingdom	28 (58.3%)	18 (47.3%)	14 (82.3%)	5 (55.6%)	65 (58.0%)
Europe	3 (6.2%)	1 (2.6%)	1 (5.8%)	3 (33.3%)	8 (7.1%)
United States	2 (4.2%)	18 (47.3%)	1 (5.8%)	1 (11.1%)	22 (19.6%)
Canada	6 (12.5%)	–	–	–	6 (5.3%)
Australia and New Zealand	2 (4.2%)	–	1 (5.8%)	–	3 (2.6%)
Asia	7 (14.5%)	1 (2.6%)	–	–	8 (7.1%)
Prior experience in...					
Designing PROs	10 (20.8%)	16 (42.1%)	4 (23.5%)	–	30 (29.1%)
1-3 trials	5 (10.4%)	11 (28.9%)	2 (11.7%)		18 (16.1%)
4-6	3 (6.2%)	3 (6.2%)	1 (5.8%)		7 (6.2%)
7-10	1 (2.1%)	1 (2.6%)	–		2 (1.8%)
>10	1 (2.1%)	1 (2.6%)	1		3 (2.6%)
Conducting PROs	27 (56.2%)	12 (31.6%)	12 (70.6%)	–	51 (49.5%)
1-3 trials	16 (33.3%)	11 (28.9%)	7 (41.1%)		34 (30.3%)
4-6	5 (10.4%)	1 (2.6%)	3 (17.6%)		9 (8.1%)
7-10	2 (4.2%)	–	1 (5.8%)		3 (2.6%)
>10	4 (8.3%)	–	1 (5.8%)		5 (4.4%)
Reporting PROs	6 (12.5%)	8 (21.1%)	1 (5.9%)	–	15 (14.6%)
1-3 trials	5 (10.4%)	7 (18.4%)	1 (5.8%)		13 (11.6%)
4-6	1 (2.1%)	–	–		1 (0.9%)
7-10	–	–	–		–
>10	–	1 (2.6%)	–		1 (0.9%)
Using PROs to select tolerable doses during a dose escalation meeting	1 (2.1%)	1 (2.6%)	1 (5.9%)	–	3 (2.6%)
Using PROs to help determine the MTD	1 (2.1%)	1 (2.6%)	1 (5.9%)	–	3 (2.6%)
Using PROs to help determine the RP2D	–	2 (5.3%)	1 (5.9%)	–	3 (2.6%)
Reviewed trials with PROs endpoints (for funding or regulatory approval)	–	–	–	5 (55.5%)	5 (55.5%)

#### Prior Experience

Most trialists reported no prior experience designing (73, 70.9%), conducting (52, 50.5%), or reporting (88, 85.4%) PROs in DFOT. Regulators’ experience in reviewing early phase oncology trials with a PRO endpoint was mixed—0 trials (2, 28.6%), 1–3 trials (2, 28.6%), 7–10 (1, 14.3%), >10 trials (2, 28.6%). All regulators (except 1) had prior experience approving an early phase oncology trial with a PRO endpoint. Two responses from funders were received. Both had never reviewed an early phase oncology trial containing PRO endpoints. However, both stated that they were more likely to fund a trial if PRO endpoints were included.

A total of 30 trialists reported prior experience designing DFOT with PROs, with the most common PROMs being the EORTC QLQ-C30 (16/30 respondents, 53.5%) and the PRO-CTCAE (12/30, 40%). In total 51 trialists reported experience conducting DFOT with PROs, with the most common PROMs being the EORTC QLQ-C30 (28/51, 54.9%) and the EORTC disease-specific modules (19/51, 37.3%). A total of 15 trialists reported prior experience reporting DFOT with PROs, with the most common PROMs being the EORTC QLQ-C30, PRO-CTCAE, and FACT modules (all 5/15, 33.3%).

#### Benefits of PRO Use

Participants strongly agreed/ agreed that PROs could provide data regarding frequency (86, 76.8%) and duration (81, 72.3%) of toxicities, especially in agents with moderate, chronic, or delayed toxicities (77, 68.8%), and identify new types of toxicities (75, 67.0%). There was variation in levels of perceived benefits among the different work roles (Table 2) and experience (Supplementary Table S2); with “providing information about the frequency of occurrence” being most similar among the trialists, and “reliably capture toxicity information from patients” most similar across levels of experience.

**Table 2.** Top 5 benefits of PROs in dose-finding oncology trials by work role (*n* (%))

	Clinician	Statistician	Trial manager/ administrator	Funder	Regulator	All
	(*N* = 48)	(*N* = 38)	(*N* = 17)	(*N* = 2)	(*N* = 7)	(*N* = 112)
Reliably capture toxicity information from patients	34 (70.8)	22 (57.9)	11 (64.7)	2 (100)	4 (57.1)	73 (65.2)
Identify new types of toxicities	34 (70.8)	23 (60.5)	12 (70.6)	1 (50)	5 (71.4)	75 (67)
Provide information about frequency of occurrence	36 (75)	29 (76.3)	13 (76.5)	2 (100)	6 (85.7)	86 (76.8)
Provide information about duration of toxicities	35 (72.9)	25 (65.8)	13 (76.5)	2 (100)	6 (85.7)	81 (72.3)
Capture moderate, chronic, or delayed toxicities	34 (70.8)	27 (71.1)	12 (70.6)	1 (50)	3(42.9)	77 (68.8)

PROs could also be used to guide the design of later phase trials. A total of 94 (83.9%) participants strongly agreed/agreed that PROs could be used to guide the development of PRO objectives or statistical plans (84, 75%) in later phase trials.

#### Barriers to PRO Use

Several barriers were identified to PRO use in DFOT. The top 5 concerns among participants were: lack of guidance regarding PRO selection (73/103, 70.9%), missing PRO data (71/103, 68.9%), and overburdening staff with PRO collection (68/103, 66.0%), patient queries (64/103, 62.1%), or data queries (61/103, 59.2%) (Supplementary Fig. S2). There was variation in the top 5 barriers by work role among trialists (Table 3). There was minimal difference in the top 5 barriers among those had prior experience using PROs versus those who did not.

**Table 3.** Top 5 barriers to PRO use in dose-finding oncology trials by work role among trialists (*n* (%))

	Clinicians		Statisticians		Trial manager/administrator	
	(*n* = 48)		(*n* = 38)		(*n* = 17)	
Rank	Barrier	Number (proportion)	Barrier	Number (proportion)	Barrier	Number (proportion)
1	Lack of guidance	41 (85.4)	Potential for missing data	31 (81.6)	Overburdening staff with PROs collection	12 (70.6)
2	Lack of access to specialist advice	37 (77.1)	Overburdening patients with PROs collection	26 (68.4)	Lack of guidance	11 (64.7)
3	Overburdening staff with patient queries	36 (75)	Overburdening staff with PROs collection	24 (63.2)	Lack of access to specialist advice	10 (58.8)
4	Overburdening staff with data queries	36 (75)	Lack of guidance	21 (55.3)	Potential for missing data	10 (58.8)
5	Overburdening patients with PROs collection	32 (66.7)	Overburdening staff with patient queries	19 (50.0)	Lack of concordance between CTCAE gradings and PROs	9 (52.9)

#### Attitudes to Using PROs to Inform Selection of Tolerable Doses and Regimens

**Figure 1.**
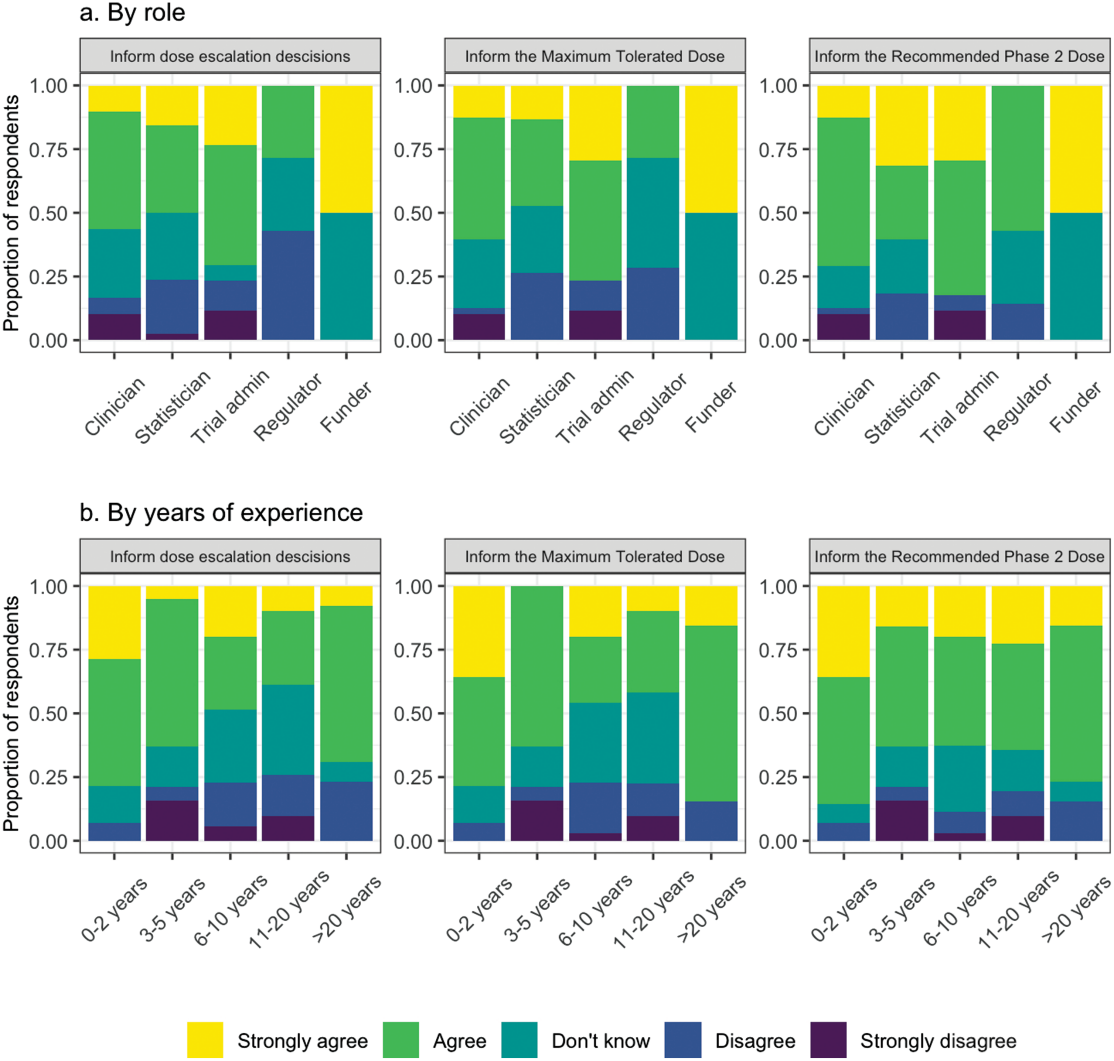
Attitudes toward PROs for dose–escalation decisions: (**a**) By work role and (**b**) by number of years of work experience.

A total of 62 (55.3%) respondents strongly agreed/agreed that PRO data on adverse events should be communicated to clinicians in real-time. Respondents agreed that PROs should be reviewed when making dose–escalation decisions (61, 54.5%), determining the MTD (63, 56.2%) and RP2D (76, 67.9%). This was despite most participants having no prior experience using PRO data to determine the MTD or RP2D (100, 97.1% for both MTD and RP2D). When this was analyzed by work role, the greatest level of agreement among the work roles was for using PROs to inform the RP2D (Fig. 1a). Those with between 6 and 20 years of experience had lower levels of agreement for using PROs to inform dose-escalation decisions and the MTD, but similar levels of agreement for using PROs to inform the RP2D compared to those with <5 years of experience (Fig. 1b). PROs were viewed mainly as a secondary (66, 58.9%) or exploratory (54, 48.2%) endpoint in DFOT.

#### Analysis of Free Text Responses

Free text responses were received from 51 participants, 41 of whom work in academia. Five themes were identified, namely, data issues, design issues, implementation issues, the role of PROs, and regulatory considerations (Supplementary Table S3).

Participants reported concerns around trial design and PRO data itself, including the potential bias resulting from patients under or over-reporting adverse events. Statisticians cited concerns about trial design features, such as small sample size and lack of randomization. Trial managers expressed concern about implementation issues including delays to a trial set up and data collection and monitoring, especially ensuring PRO data on side effects were reviewed in a timely manner. Among regulators, there was no consensus as to whether the use of PROs would influence decision-making; nearly all respondents stated that it would depend on different aspects such as clinical value and data reliability.

### Survey of the NCRI Consumer Forum

A total of 57 responses were received. Most (53, 93.0%) had not used PROs in an oncology drug trial. Most were willing to spend ≤ 15 min/day completing PROs (*n* = 28, 49.1%). 55 (96.5%) participants preferred to complete PROs electronically.

**Figure 2.**
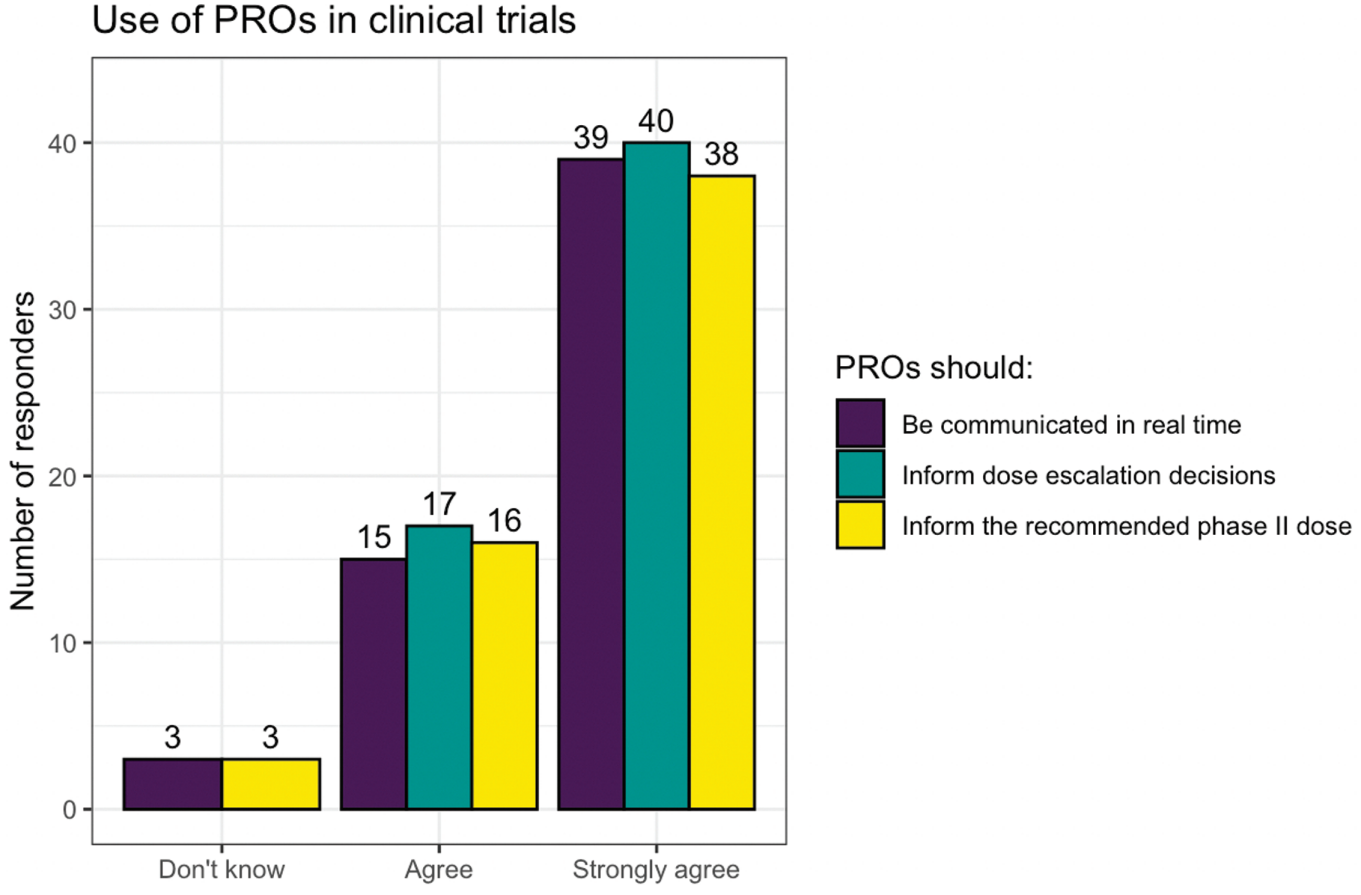
NCRI consumer forum—attitudes toward using PROs in dose-finding oncology trials.

**Figure 3.**
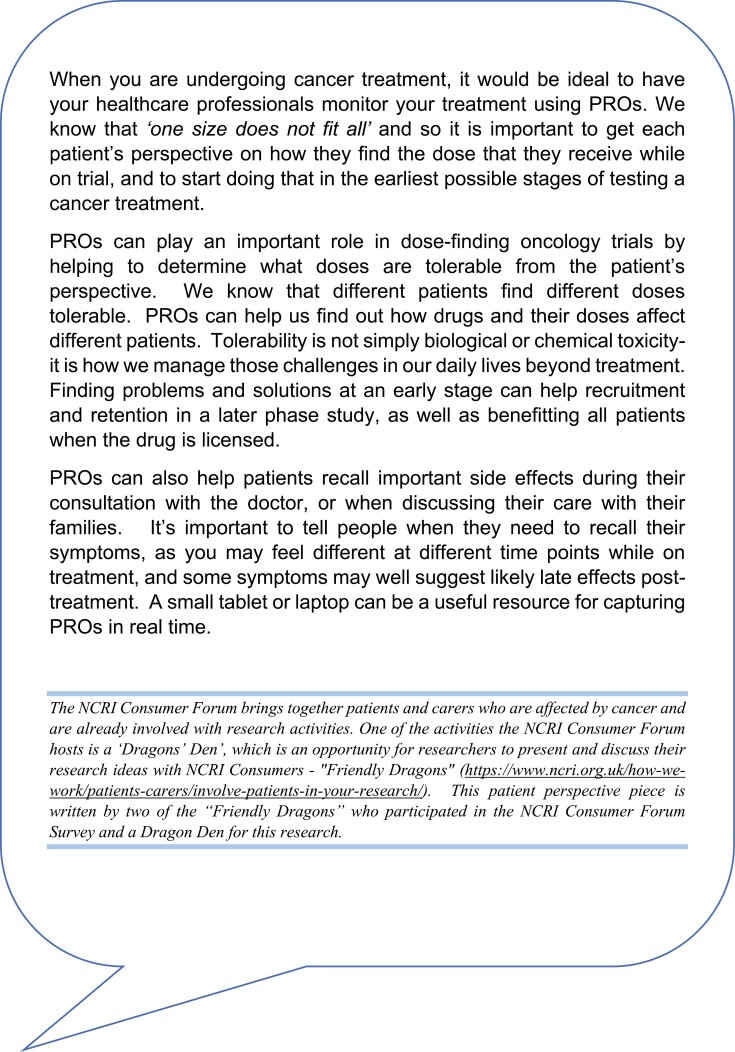
NCRI consumer forum—a consumer perspective (Della Ogunleye and Richard Stephens).

Most participants strong agreed/agreed that PROs could provide useful information for an individual patient’s care (*n* = 53, 93.0%) or for dose-finding trials (*n*= 54, 94.7%). Most participants strongly agreed/agreed that PROs should be communicated in real-time to clinicians, and inform dose–escalation decisions and the RP2D (Figs. 2 and 3).

## Discussion

These surveys provide critical insights into the attitudes of trialists and consumers toward the inclusion and use of PROs in DFOT.

Most trialists reported minimal prior experience designing, conducting, or analyzing DFOT with PRO endpoints, which is consistent with the limited number of trials with PRO endpoints registered on ClinicalTrials.gov. Despite this, trialists were keen to explore ways PROs could contribute to identifying tolerable doses and regimens. The greatest degree of support was seen for using PROs to define the RP2D, suggesting that PROs may be best collected just prior to the commencement of later phase studies, as it could then be used to assess the feasibility of PRO assessment and inform sample size calculations for later phase studies. In contrast, in the NCRI Consumer Forum survey, over 90% of participants strongly supported using PROs to inform dose-escalation decisions as well as the RP2D. Patients also strongly supported feeding PROs back to clinicians in real-time to inform clinical care. Ensuring PRO systems can perform both these roles will be crucial for engaging patients and clinicians in PRO collection in DFOT.

Several benefits of using PROs were identified by trialists, including using PROs to provide data on new types of toxicities, and the frequency and duration of toxicities. This is particularly important at a time when novel anti-cancer therapies may produce moderate or delayed toxicities, which may not be adequately captured by standard methods of assessing toxicities during a DLT period. Of note, the greatest area of agreement among trialists was using PROs to characterize the frequency of toxicities, indicating that many believe this could be improved in current adverse event reporting in DFOT.

Several barriers were also identified. Some have been previously described including a lack of guidance about PRO selection, lack of experience/training in using PROs, overburdening patients and staff with PRO collection,^20,24^ dealing with missing data, and difficulties publishing PRO findings.^25,26^ Strategies to manage these issues include electronic PRO collection to minimize data entry, increase data accuracy, and reduce missing data and long-term costs.^24,27-29^ Electronic PROs may also improve the efficiency during the clinical encounter by promptly identifying adverse events rather than needing to collect this data during the interview. The feasibility and acceptability of collecting electronic PROs in DFOT have already been demonstrated in a single centre Canadian study.^21^ Real-time alerts based on pre-defined algorithms can assist with real-time monitoring and early management of adverse events.^30^ Further work is required to explore whether these strategies are acceptable to stakeholders in DFOT. Obtaining funding for electronic PRO collection may be challenging, particularly for academic-sponsored studies. The seamless integration of PRO collection systems with multiple data entry platforms for DFOT may also be difficult. Careful selection of PRO instruments and the timing and frequency of assessments are also important to minimize the patient burden. While the NCRI survey suggested that spending *≤* 15 min per day on PROs was acceptable, more detailed feedback will be critical in future feasibility studies.

Other barriers were unique to the DFOT setting, including the potential discordance between PRO data and clinician-assessed AEs which could affect trial integrity, PROs not providing any additional information compared to CTCAE gradings, and inadequate time to add PROs when planning a DFOT. Concerns related to over- or under-reporting of adverse events were also expressed, although it is well-recognized that this is also an issue with clinician-assessed CTCAE gradings.^2,31^ Similarly, concerns about the potential for patient bias in PRO reporting in open-label DFOT were also expressed, although recent data from Efficace et al supports the validity of PROs in open-label trials.^32^

The international survey of trialists has several strengths. It was representative of the early phase oncology workforce, with a mix of work roles, levels of experience, and countries, ensuring a diversity of opinions was captured. The survey was anonymized, ensuring participants were able to provide honest responses without concern that their responses could be attributed to themselves or their institution.

The limitations of this study were that the survey was voluntary and therefore may be biased toward those who had an interest in the topic. Funders and regulators, as well as trialists from the pharmaceutical industry, were under-represented and further work is needed to capture these opinions. Whilst this study included participants from a variety of countries, participants were predominantly from the United Kingdom and the United States. Future work should focus on obtaining perspectives from under-represented countries to encourage the inclusion of PROs in DFOT globally.

The NCRI Consumer Forum includes consumers and their carers from a diverse range of cultural backgrounds. The Forum survey, therefore, provides data on consumers’ and carers’ perspectives on using PROs in DFOT, highlighting key priorities for using PROs in this setting including as part of clinical care. However, consumers did not need to have experienced an early phase trial to be eligible. More data focusing on consumers who have participated in early phase trials from a more diverse range of countries and backgrounds are needed to understand the unique challenges faced by this patient group.

Further work is required to understand the optimal settings in which to use PRO data in DFOT.^33^ During the clinical encounter, PRO data could be shared with clinicians to inform CTCAE gradings or collected separately and not shared with clinicians in real-time. Following the clinical encounter, PRO data could be reviewed at the time of dose escalation decisions or selection of the MTD or RP2D alongside other clinical, pharmacological and pharmacodynamic data, or it could be reviewed once a dose has been selected to confirm it is tolerable. Each of these options has differing workload implications and would need a thorough testing in any future trials. For example, if PROs were collected separately from clinician-assessed adverse events, work would be needed to determine how best to reconcile clinician and patient reports and determine causation. This could result in additional data queries, overburdening of staff, and delays in trial progress. However, Basch et al argue that patient-reported adverse events are complementary to clinician-reported adverse events, and it is necessary to collect both to comprehensively evaluate the toxicities of novel drugs.^34^ It would also be critical to inform patients how their data will be used, and if/when it will be reviewed by the clinical team to ensure patient safety during study.

Future guidelines should provide guidance regarding PRO selection in DFOT when toxicities may not be entirely known and setting standards for analyzing and reporting PRO data for publication.

## Conclusion

Patient-reported adverse events are increasingly regarded as valuable and complementary to clinician-reported adverse events. We identified that whilst patients and trialists have minimal experience using PROs in DFOT, there is broad support for using PROs to inform the selection of tolerable doses and regimens. However, there are several barriers to uptake and use in this setting. Further collaboration between international stakeholders is needed to inform the research agenda in this area. Guidelines are needed to standardize the selection, analysis, and reporting of PROs and to increase the efficiency of PRO collection in DFOT.

## Supplementary Material

oyac117_survey_Supplementary_Figures

oyac117_survey_Supplementary_Tables

## Data Availability

De-identified participant data underlying the results reported in this article will be shared. Data will be available immediately following publication with no end date. Investigators who propose the use of the data that has been approved by an independent review committee will be granted access to the data to achieve the aims of the approved proposal. Proposals should be directed to Christina.Yap@icr.ac.uk.
