# Supplementary material for: Using Patient-Reported Outcomes in Dose-Finding Oncology Trials: Surveys of Key Stakeholders and the National Cancer Research Institute Consumer Forum
Source: Oncologist. 2022 Jun 28;27(9):768–77. doi: 10.1093/oncolo/oyac117 (PMC9438918; doi:10.1093/oncolo/oyac117)

Supplementary Fig. S1: Survey map

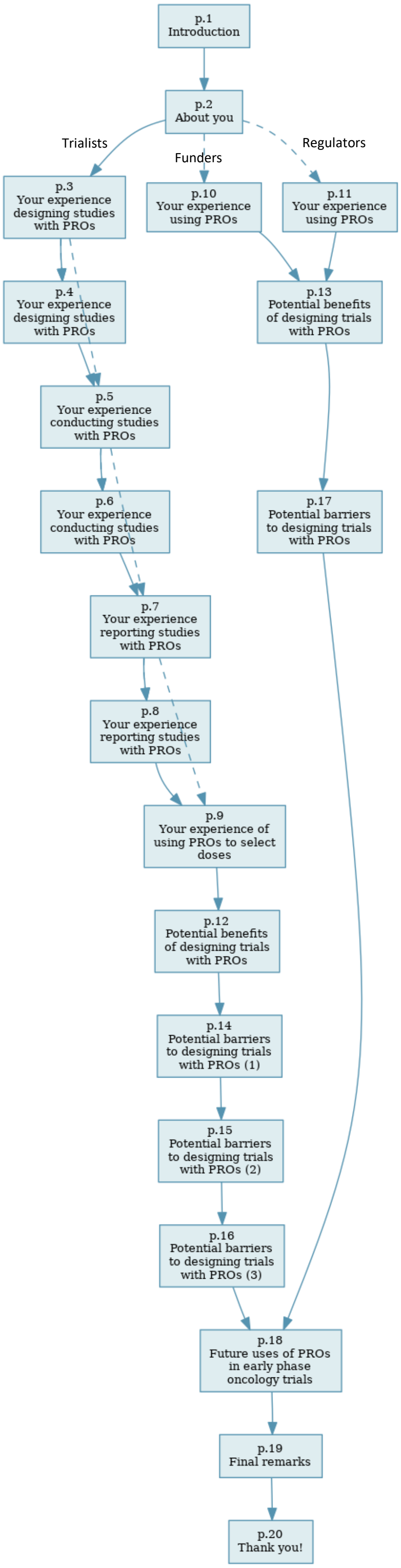

Supplementary Fig. S2: Top 10 barriers according to clinicians, statisticians and trial managers/ administrators

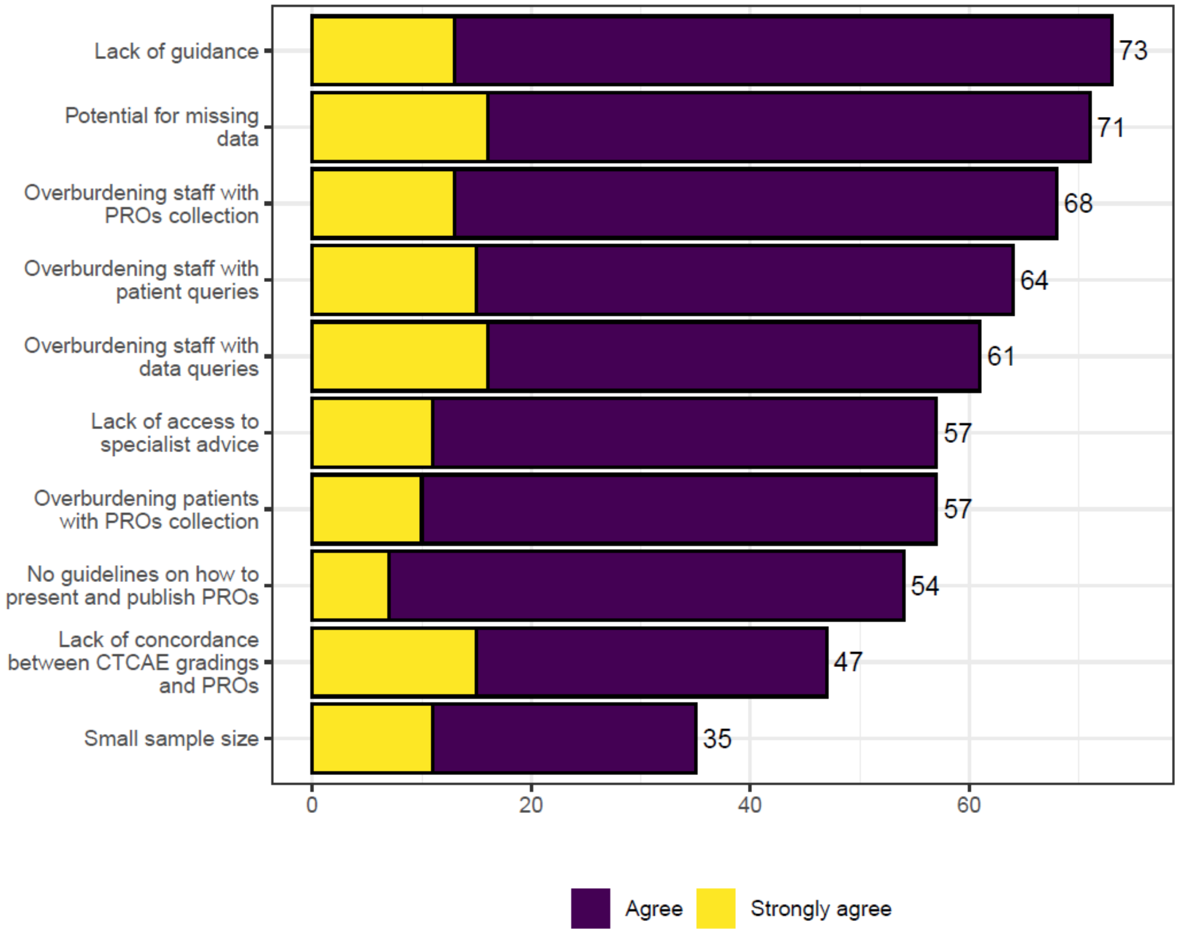

Supplement: oyac117_survey_Supplementary_Figures [file oyac117_survey_supplementary_figures.pdf]
