# Supplementary material for: Using Patient-Reported Outcomes in Dose-Finding Oncology Trials: Surveys of Key Stakeholders and the National Cancer Research Institute Consumer Forum
Source: Oncologist. 2022 Jun 28;27(9):768–77. doi: 10.1093/oncolo/oyac117 (PMC9438918; doi:10.1093/oncolo/oyac117)
Supplement: oyac117_survey_Supplementary_Tables [file oyac117_survey_supplementary_tables.pdf]

Assessing stakeholder attitudes towards incorporating Patient Reported Outcomes (PROs) into early phase oncology trials

Introduction

Patient-Reported Outcomes (PROs) are increasingly being incorporated into oncology trials. They are defined as ‘any report of the status of a patient’s health condition that comes directly from the patient, without interpretation of a patient’s response by a clinician or anyone else’. This can include patient-reported symptoms (eg. fatigue, pain), functional outcomes (eg. physical, emotional, social functioning) or multi-dimensional constructs (eg. health-related quality of life). Examples of questionnaires (or measures) used to collect PROs include the EORTC QLQ C30 and the FACT-G.

PROs are collected widely in later phase clinical trials and are increasingly recognised as valuable by patients, clinicians, funders and regulators. However, the collection and analysis of PRO data in early phase trials is limited.

We are a group of clinicians and statisticians from the Institute of Cancer Research and the Royal Marsden Hospital in London, United Kingdom, interested in understanding people’s attitudes towards using PROs in early phase oncology trials. ***In this survey, we refer to early phase oncology trials as phase I and phase I/II trials with a dose finding component*.** This survey will help to shape our future use of PROs in early phase trials.

What is involved?

The survey should take 10 minutes to complete. You will have the option to complete

the survey in one sitting, or save your responses and complete it at another time by clicking on 'Finish later'.

This survey is voluntary. No identifiable data will be collected, so comments will not be attributable to any individual or organisation. All collected data will be used for non-commercial research activities only. This study has been approved by the Committee for Clinical Research at the Royal Marsden Hospital, United Kingdom.

The findings of this survey will be presented and published.

Access to the survey will close on **30 November 2020**.

About you

Please select the statement that best describes your current work role: ✱ *Required*

- Clinician working in a hospital or academia as a Principal Investigator
- Clinician working in a hospital or academia as a Sub-Investigator
- Clinician working in a pharmaceutical/ biotechnology setting
- Trial manager/administrator working in a hospital or academia (eg. study managers, clinical research associates)
- Trial manager/ administrator working in a pharmaceutical/ biotechnology setting (eg. study managers, clinical research associates)
- Statistician working in a hospital or academia
- Statistician working in a pharmaceutical/ biotechnology setting
- Funder
- Regulator

How long have you worked in early phase oncology trials? ✱ *Required*

- 0-2 years
- 3-5 years
- 6-10 years
- 11-20 years
- >20 years

Where is your current primary place of work? ✱ *Required*

- United Kingdom
- Europe
- United States

- Canada
- Asia
- Australia or New Zealand
- Africa
- Other

If you selected Other, please specify:

Your experience designing studies with PROs

Have you ***designed*** an early phase oncology trial (phase 1 or phase 1/2 trial with a dose finding component) that collected or is collecting PROs? ✱ *Required*

- ◯ Yes
- ◯ No

Your experience designing studies with PROs

How many studies? ✱ *Required*

- 1-3
- 4-6
- 7-10
- >10

What phase were these studies (choose all that apply)? ✱ *Required*

- Phase 1 dose escalation
- Phase 1 dose escalation and expansion
- Phase 1/2

What PRO measures were used (choose all that apply)? ✱ *Required*

- EORTC generic modules (eg. EORTC QLQ C30)
- EORTC disease-specific modules (eg. Breast, colorectal, lung)
- FACT measures
- PROMIS measures
- Patient-reported outcome- CTCAE (PRO-CTCAE)
- Uncertain
- Other

If you selected Other, please specify:

Your experience conducting studies with PROs

Have you been involved in the ***conduct*** of an early phase oncology trial (phase 1 or phase 1/2 trial with a dose finding component) that collected PRO data? ✱ *Required*

- Yes
- No

Your experience conducting studies with PROs

How many studies? ✱ *Required*

- 1-3
- 4-6
- 7-10
- >10

What phase were these studies (choose all that apply)? ✱ *Required*

- Phase 1 dose escalation
- Phase 1 dose escalation and expansion
- Phase 1/2

What PRO measures were used (choose all that apply)? ✱ *Required*

- EORTC generic modules (eg. EORTC QLQ C30)
- EORTC disease-specific modules (eg. Breast, colorectal, lung)
- FACT measures
- PROMIS measures
- Patient-reported outcome- CTCAE (PRO-CTCAE)
- Uncertain
- Other

If you selected Other, please specify:

Your experience reporting studies with PROs

Have you been involved in the ***reporting*** (analysis and/or interpretation) of an early phase oncology trial (phase 1 or phase 1/2 trial with a dose finding component) that collected PRO data? ✱ *Required*

- Yes
- No

Your experience reporting studies with PROs

How many studies? ✱ *Required*

- ◯ 1-3
- ◯ 4-6
- ◯ 7-10
- ◯ >10

What phase were these studies (choose all that apply)? ✱ *Required*

- ☐ Phase 1 dose escalation
- ☐ Phase 1 dose escalation and expansion
- ☐ Phase 1/2

What PRO measures were used (choose all that apply)? ✱ *Required*

- ☐ EORTC generic modules (eg. EORTC QLQ C30)
- ☐ EORTC disease-specific modules (eg. Breast, colorectal, lung)
- ☐ FACT measures
- ☐ PROMIS measures
- ☐ Patient-reported outcome- CTCAE (PRO-CTCAE)
- ☐ Uncertain
- ☐ Other

If you selected Other, please specify:

How was the PRO data analysed? ✱ *Required*

Please select at least 1 answer(s).

- [ ] Comparison between specific time points eg. before versus after treatment
- [ ] Longitudinal analysis over time eg. analysing trends in adverse events or health-related quality of life over multiple time points
- [ ] Don't know
- [ ] Other

If you selected Other, please specify:

Your experience of using PROs to select doses

Have you ever used PRO data to help ***select tolerable doses*** during a ***dose escalation*** meeting? ✱ *Required*

- ○ Yes
- ○ No

If yes, please briefly describe the study and describe how the PRO data was used to select tolerable doses.

Have you ever used PRO data to help ***determine the Maximum Tolerated Dose***? ✱ *Required*

- ○ Yes
- ○ No

If yes, please briefly describe the study and how the PRO data was used to determine the Maximum Tolerated Dose.

Have you ever used PRO data to help ***determine the Recommended Phase 2 Dose***?

✱ *Required*

- Yes
- No

If yes, please briefly describe the study and how the PRO data was used to determine the Recommended Phase 2 Dose.

Your experience using PROs

Please estimate of the number of early phase oncology trials (phase 1 or phase 1/2 trials with a dose finding component) you have ***reviewed*** for funding that contained PRO endpoints. ✱ *Required*

- 0
- 1-3
- 4-6
- 7-10
- >10

When reviewing an early phase oncology study for funding, are you ***more or less likely*** to fund a study that includes PRO endpoints? ✱ *Required*

- More likely to fund
- Less likely to fund
- Uncertain

Please provide further detail regarding your response.

Your experience using PROs

Please estimate the number of early phase oncology trials (phase 1 or phase 1/2 trials with a dose finding component) you have ***reviewed for drug approval*** that contained PRO endpoints. ✱ *Required*

- 0
- 1-3
- 4-6
- 7-10
- >10

Please estimate the number of early phase oncology trials you have approved that contained PROs. ✱ *Required*

- 0
- 1-3
- 4-6
- 7-10
- >10

When reviewing an early phase oncology study, are you more or less likely to approve a drug that includes PRO endpoints? ✱ *Required*

- More likely to approve
- Less likely to approve
- Uncertain

Please provide further details regarding your response.

Potential benefits of designing trials with PROs

Read the following statements about the ***potential benefits*** of incorporating PROs into early phase trials (phase 1 or phase 1/2 trials with a dose finding component). Select the rating that best applies to you. ✱ *Required*

Please don't select more than 1 answer(s) per row.

Please select at least 10 answer(s).

	Strongly disagree	Disagree	Don't know	Agree	Strongly agree
PROs are a RELIABLE method of capturing information about toxicities from a patient's perspective.	☐	☐	☐	☐	☐
PROs highlight NEW TYPES OF TOXICITIES not captured by clinician-assessed CTCAE gradings.	☐	☐	☐	☐	☐
PROs provide additional information regarding the FREQUENCY of toxicities.	☐	☐	☐	☐	☐
PROs provide additional information regarding the DURATION of toxicities.	☐	☐	☐	☐	☐

PROs are useful for capturing toxicities in classes of drugs with MODERATE, CHRONIC toxicities or DELAYED side effects eg. immunotherapy.	☐	☐	☐	☐	☐
PRO data should be reviewed when making DOSE ESCALATION decisions.	☐	☐	☐	☐	☐
PRO data should be reviewed when determining the MAXIMUM TOLERATED DOSE.	☐	☐	☐	☐	☐
PRO data should be reviewed when determining the RECOMMENDED PHASE 2 DOSE.	☐	☐	☐	☐	☐
Collecting PROs during Phase 1 can help GUIDE the development of PRO OBJECTIVES in later phase studies.	☐	☐	☐	☐	☐

Collecting PROs during Phase 1 can help guide STATISTICAL PLANNING for PRO endpoints in later phase studies.	☐	☐	☐	☐	☐

Potential benefits of designing trials with PROs

Read the following statements about the ***potential benefits*** of incorporating PROs into early phase trials (phase 1 or phase 1/2 trials with a dose finding component). Select the rating that best applies to you.

Please don't select more than 1 answer(s) per row.

Please select at least 10 answer(s).

	Strongly disagree	Disagree	Don't know	Agree	Strongly agree
PROs are a RELIABLE method of capturing information about toxicities from a patient’s perspective.	☐	☐	☐	☐	☐
PROs highlight NEW TYPES OF TOXICITIES not captured by clinician-assessed CTCAE gradings.	☐	☐	☐	☐	☐
PROs provide additional information regarding the FREQUENCY of toxicities.	☐	☐	☐	☐	☐
PROs provide additional information regarding the DURATION of toxicities.	☐	☐	☐	☐	☐

PROs are useful for capturing toxicities in classes of drugs with MODERATE, CHRONIC toxicities or DELAYED side effects eg. immunotherapy.	☐	☐	☐	☐	☐
PRO data should be reviewed when making DOSE ESCALATION decisions.	☐	☐	☐	☐	☐
PRO data should be reviewed when determining the MAXIMUM TOLERATED DOSE.	☐	☐	☐	☐	☐
PRO data should be reviewed when determining the RECOMMENDED PHASE 2 DOSE.	☐	☐	☐	☐	☐
Collecting PROs during Phase 1 can help GUIDE the development of PRO OBJECTIVES in later phase studies.	☐	☐	☐	☐	☐

Collecting PROs during Phase 1 can help guide STATISTICAL PLANNING for PRO endpoints in later phase studies.	☐	☐	☐	☐	☐

Potential barriers to designing trials with PROs (1)

Read the following statements about potential barriers to incorporating PROs into early phase trials. Select the rating that best applies to you. ✱ *Required*

Please don't select more than 1 answer(s) per row.

Please select at least 8 answer(s).

	Strongly disagree	Disagree	Don't know	Agree	Strongly agree
I do not have any personal EXPERIENCE in using PROs and are therefore not comfortable incorporating them into new trials.	☐	☐	☐	☐	☐
I have not received any formal TRAINING in using PROs and are therefore not comfortable incorporating them into new trials.	☐	☐	☐	☐	☐
I am concerned about the lack of GUIDANCE regarding what PROs to select, and how to incorporate them, into early phase oncology trials.	☐	☐	☐	☐	☐

I am concerned about the lack of access to SPECIALIST ADVICE (eg. statisticians or PRO experts) regarding PRO selection and study design.	☐	☐	☐	☐	☐
There is inadequate TIME during study design to consider including PRO endpoints.	☐	☐	☐	☐	☐
In my experience, FUNDERS do not respond positively to the increased costs associated with PRO collection.	☐	☐	☐	☐	☐
PROs do not provide any ADDITIONAL INFORMATION to clinician-assessed CTCAE gradings regarding the toxicity of novel agents.	☐	☐	☐	☐	☐

I am concerned there may be a LACK OF CONCORDANCE between patient-assessed toxicities and clinician-assessed toxicities which could affect trial integrity.	☐	☐	☐	☐	☐

Potential barriers to designing trials with PROs (2)

Read the following statements about potential barriers to incorporating PROs into early phase trials. Select the rating that best applies to you. ✱ *Required*

Please don't select more than 1 answer(s) per row.

Please select at least 7 answer(s).

	Strongly disagree	Disagree	Don't know	Agree	Strongly agree
I am concerned about overburdening PATIENTS with PRO collection.	☐	☐	☐	☐	☐
I am concerned about overburdening trial STAFF with PRO collection and analysis.	☐	☐	☐	☐	☐
I am concerned about overburdening trial staff/ nursing staff with PATIENT QUERIES generated by reporting PROs.	☐	☐	☐	☐	☐
I am concerned about overburdening trial staff with DATA QUERIES generated by collecting PROs.	☐	☐	☐	☐	☐

I am concerned regarding the lack of access to an ELECTRONIC PRO system for PRO collection in my institution (and believe paper/ telephone assessment is not feasible).					
The COST of PRO collection is prohibitive to a trial.					
The COST of analysing PRO data is prohibitive to a trial.					

Potential barriers to designing trials with PROs (3)

Read the following statements about potential barriers to incorporating PROs into early phase trials. Select the rating that best applies to you. ✱ *Required*

Please don't select more than 1 answer(s) per row.

Please select at least 5 answer(s).

	Strongly disagree	Disagree	Don't know	Agree	Strongly agree
I am concerned about the potential for MISSING PRO DATA and therefore the ability to make sense of the results.	☐	☐	☐	☐	☐
PRO outcomes will not be useful as SAMPLE SIZES are small in early phase oncology trials.	☐	☐	☐	☐	☐
I am concerned that PRO results will not be considered in the DRUG APPROVAL process and therefore do not think it is worthwhile.	☐	☐	☐	☐	☐

I am concerned about the lack of guidelines as to how to PRESENT and PUBLISH PRO findings from early phase trials.	☐	☐	☐	☐	☐
I am concerned that JOURNALS will not be interested in publishing PRO results from early phase trials.	☐	☐	☐	☐	☐

Potential barriers to designing trials with PROs

Read the following statements about potential barriers to incorporating PROs into early phase trials. Select the rating that best applies to you.

Please don't select more than 1 answer(s) per row.

Please select at least 5 answer(s).

	Strongly disagree	Disagree	Don't know	Agree	Strongly agree
I am concerned about the potential for MISSING PRO DATA and therefore the ability to make sense of the results.	☐	☐	☐	☐	☐
PRO outcomes will not be useful as SAMPLE SIZES are small in early phase oncology trials.	☐	☐	☐	☐	☐
I am concerned that PRO results will not be considered in the DRUG APPROVAL process and therefore do not think it is worthwhile.	☐	☐	☐	☐	☐

I am concerned about the lack of guidelines as to how to PRESENT and PUBLISH PRO findings from early phase trials.	☐	☐	☐	☐	☐
I am concerned that JOURNALS will not be interested in publishing PRO results from early phase trials.	☐	☐	☐	☐	☐

Future uses of PROs in early phase oncology trials

There is increasing interest in incorporating PRO data into traditional early phase endpoints, such as Maximum Tolerated Dose or Recommended Phase 2 Dose. However, the utility and methodology for doing so is unclear.

Read the following statements regarding potential ways in which PRO data could be used to inform traditional early phase endpoints. The following statements refer to the use of PROs in ***dose escalation*** studies. Select the rating that best applies to you. ✱ *Required*

Please don't select more than 1 answer(s) per row.

Please select at least 3 answer(s).

	Strongly disagree	Disagree	Don't know	Agree	Strongly agree
PRO data on adverse events should be communicated to investigators in real-time to inform their CTCAE gradings	☐	☐	☐	☐	☐
PRO data on adverse events (IN CONJUNCTION WITH clinician-assessed CTCAE data) should be used to inform dose escalation decisions.	☐	☐	☐	☐	☐

PRO data on adverse events (IN CONJUNCTION WITH clinician-assessed CTCAE data) should be used to inform the Maximum Tolerated Dose.	☐	☐	☐	☐	☐
PRO data should only be reviewed once the Maximum Tolerated Dose has been determined.	☐	☐	☐	☐	☐

When should PRO data be reviewed in the ***dose escalation meeting***? ✱ *Required*

- ○ PRO data should be presented alongside clinician-assessed CTCAE gradings BEFORE a dose is deemed tolerable.
- ○ PRO data should be reviewed AFTER a dose has been selected to confirm whether the dose is tolerable.
- ○ Neither- PROs should not be reviewed

Read the following statements regarding the use of PRO data in the ***dose expansion*** setting. Select the rating that best applies to you. ✱ *Required*

Please don't select more than 1 answer(s) per row.

Please select at least 1 answer(s).

	Strongly disagree	Disagree	Don't know	Agree	Strongly agree

PRO data on adverse events (IN CONJUNCTION WITH CTCAE data) should be used to determine the Recommended Phase 2 Dose.	☐	☐	☐	☐	☐

When should PRO data be reviewed in the dose expansion setting? ✱ *Required*

- ○ PRO data should be considered alongside clinician-assessed CTCAE gradings BEFORE a dose is selected as the Recommended Phase 2 dose.
- ○ PRO data should be reviewed AFTER a Recommended Phase 2 Dose has been selected to confirm whether or not a dose is tolerable.
- ○ Neither- PROs should not be reviewed

Where would you expect to see PROs included as a ***trial endpoint*** in early phase oncology trials? (choose all that apply) ✱ *Required*

- ☐ Co-primary endpoint
- ☐ Secondary endpoint
- ☐ Exploratory endpoint

Do you have any other concerns about using PROs to guide selection of tolerable doses? ✱ *Required*

- ○ Yes
- ○ No

If yes, please provide further details.

Final remarks

Is there anything else you wish to tell us about using PROs in early phase oncology trials?

Thank you!

Thank you for participating in the survey!

Please feel free to share this survey with any interested colleagues.

https://icr.onlinesurveys.ac.uk/prosinearlyphaseoncologytrials

We look forward to sharing the results with you in the near future.

Supplementary Table S1: CHERRIES checklist

	Checklist item	Description
Design	Describe survey design	A prospective, single time point, global online survey
IRB approval and informed consent	Mention whether the study has been approved by an IRB	Following review by the Royal Marsden Hospital Service Evaluation Committee, the study was deemed exempt from full review and approval by a research ethics committee as a Service Evaluation as per HRA Guidance 2016. It was approved by the Royal Marsden Hospital Committee for Clinical Research (SE983 dated 23/7/2020)
	Informed consent process	The first page of the survey provided background information, aims of the survey, expected completion time, and closure data. It stated that the survey was voluntary, that no identifiable data would be collected, and that collected data would be used for research purposes only.<br>It did not specifically explain how long the data would be kept.<br>Informed consent was inferred by participants clicking on the 'next' button to start the survey.
	Data protection	No identifiable information was collected or stored.
Development and pre-testing	Development and testing	The survey was developed by the study authors.<br>Survey content was determined through a literature review and review of current PRO use in early phase oncology trials on ClinicalTrials.gov [36,37]<br>The usability and technical functionality tested by several ICR staff members prior to deployment.
Recruitment process and description of sample	Open vs closed survey	Open to targeted participants and all those in networks to which it was distributed. Potential participants were emailed a link to the survey. The survey was not open to the public.
	Contact mode	Initial contact with potential participants was made via email.
	Advertising the survey	The surveys were announced via the mailing lists of the Experimental Cancer Medicine Centres UK, Association of British Pharmaceutical Industry, NIHR Statistics Group, American Society of Clinical Oncology, National Cancer Research Institute UK, PROTEUS consortium, and the New South Wales Early Phase Clinical Trials Alliance using a standardised email.<br>Funders approached via standardised email were Cancer Research UK, Cancer Research Wales, Pancreatic Cancer UK, Blood Cancer UK, Prostate Cancer Research Centre, Brain Tumour Research, Breast Cancer Now, Tenovus, Children with Cancer UK and Prostate Cancer UK.<br>Regulators approached via standardised email were the USA Food and Drug Administration, the European Medicines Agency and the Medicines and Healthcare Products Regulatory Agency UK.<br>Personal contacts of the authors were also contacted via email.
Survey administration	Web/email	The survey was distributed via email. Within the email, potential participants were provided with a generic link to the survey.
	Context	The questionnaire was hosted by JISC online surveys. The survey was not posted on any public websites. The JISC online survey platform also provides an option to 'discourage search engines' and this was selected.
	Mandatory/ voluntary	The survey was voluntary.
	Incentives	No incentives were provided.
	Time/date	Data was collected from 14/9/2020- 30/11/2020.
	Randomisation of items	Randomisation of questions was not performed. All participants answered the questionnaire in the same order.
	Adaptive questioning	Adaptive questioning was used. Participants completed different parts of the questionnaire according to their work role and their responses to certain questions. A survey map is provided in Appendix 2.
	Number of items	The questionnaire contained 35 questions. However, as adaptive questioning was used, participants completed a varying number of questions according to their work role and previous responses.<br>1 question was asked per page. However, some questions required multiple responses.
	Completeness checker	All items (according to work role and previous responses) were mandatory to ensure completion.
	Review step	Respondents were not able to review or change their answers.
Response rates	Unique site visitor	The questionnaire did not determine if visitors to the site were unique.
	View rate (ratio of unique survey visitors/ unique site visitors)	The view rate was not calculated as number of unique site visitors were not captured<br>858 views were recorded for the first introductory page of the survey.
	Participation rate (ratio of unique visitors who agreed to participate/ unique first survey page visitors)	The participation rate was not calculated as number of unique site visitors were not captured.
	Completion rate (ratio of users who completed the survey/ users who agreed to participate)	The completion rate was 56.6%.<br>(112 complete responses were received. It is assumed that 198 agreed to participate as they proceeded to the second survey page to start the survey.)

Preventing multiple entries from the same individual	Cookies used	JISC Online Surveys does not use cookies to assess multiple entries from the same individual to maintain participant anonymity. There is also little incentive for multiple entries from the same individual, including no remuneration.
	IP check	JISC Online Surveys does not collect information regarding respondent's IP addresses to maintain participant anonymity.
	Log file analysis	No techniques were used to analyze the log file for identification of multiple entries.
	Registration	Unique logins were not required to access the survey.
Analysis	Handling of incomplete questionnaires	As all questions were mandatory, there were no incomplete questions. If the user terminated the survey early, these responses were not analysed.
	Questionnaires submitted with an atypical timestamp	The time taken to complete the questionnaire was measured. Participants were allowed as much time as needed to complete the questionnaire.
	Statistical correction	No statistical corrections were used eg. Weighting of items or propensity scores.

Supplementary Table S2: Participant agreement (n (%)) about the potential benefits of PROs in DFOT

A: By number of years of experience

	**0-2 years (N=14)**	**3-5 years (N=19)**	**6-10 years (N=35)**	**11-20 years (N=31)**	**>20 years (N=13)**	**All (N=112)**
Reliably capture toxicity information from patients	9 (64.3)	13 (68.4)	23 (65.7)	19 (61.3)	9 (69.2)	73 (65.2)
Identify new types of toxicities	11 (78.6)	13 (68.4)	23 (65.7)	19 (61.3)	9 (69.2)	75 (67)
Provide information about frequency of occurrence	13 (92.9)	14 (73.7)	27 (77.1)	22 (71)	10 (76.9)	86 (76.8)
Provide information about duration of toxicities	12 (85.7)	15 (78.9)	25 (71.4)	19 (61.3)	10 (76.9)	81 (72.3)
Capture moderate, chronic, or delayed toxicities	10 (71.4)	15 (78.9)	24 (68.6)	19 (61.3)	9 (69.2)	77 (68.8)

B: By prior experience designing, conducting or reporting PROs in DFOT (excluding funders and regulators)

	Has prior experience (n=60)		No prior experience (n=43)		Total (n=103)	
**Rank**	**Benefit**	**Number (%)**	**Benefit**	**Number (%)**	**Benefit**	**Number (%)**
1	Provide information about duration of toxicities	44 (73.3)	Provide information about frequency of occurrence	35 (81.4)	Provide information about frequency of occurrence	78 (75.7)
2	Provide information about frequency of occurrence	43 (71.7)	Capture moderate, chronic, or delayed toxicities	35 (81.4)	Provide information about duration of toxicities	73 (70.9)
3	Identify new types of toxicities	38 (63.3)	Reliably capture toxicity information from patients	31 (72.1)	Capture moderate, chronic, or delayed toxicities	73 (70.9)
4	Capture moderate, chronic, or delayed toxicities	38 (63.3)	Identify new types of toxicities	31 (72.1)	Identify new types of toxicities	69 (67)
5	Reliably capture toxicity information from patients	36 (60)	Provide information about duration of toxicities	29 (67.4)	Reliably capture toxicity information from patients	67 (65)

Supplementary Table S3: Qualitative Analysis of Free Text Responses, Global Stakeholder Survey

Themes	Sub-themes	Description	Substantive quote
**Data issues**	Data reliability	The reliability of PROs data was a notable concern, specifically as it related to the data quality, population and patient-induced bias, and missing data. Eleven participants discussed the bias that may be introduced by a "poorly population", lack of compliance or over- or under-reporting of adverse events.	*"My main worry is that patients with a disposition to report any discomfort may dominate patients who are more likely to 'down-play' any adverse events they may be reporting. This could impact both the reporting and grading of [adverse] events."* (Statistician, 3-5 years experience)
	Clinical utility and data interpretation	Participants expressed that the translation between PROs data and clinical utility is unclear. The subjectivity of PROs can introduce confusion and brings into question the impact that PROs should have on treatment decisions. Two participants noted that there may be other measures of tolerability that can be used, one of whom cited treatment compliance.	*"They are not objective, as such any value function incorporating both CTCAE and PROs would likely be dominated by CTCAE."* (Statistician, 6-10 years experience)<br><br>*"My experience of patient-reported outcome (PRO) in early phase trials have some significant inconsistencies in patient subjective assessment and description compared to objective CTCAE reporting."* (Clinician, 6-10 years experience)
**Design issues**	Trials design	Statisticians were largely concerned about the trial design itself and the inferences that can be drawn from a small sample size, a short toxicity observation window (late-onset toxicities would not be captured), and the lack of randomization to a comparator group. Other design issues highlighted include a lack of historical experience with using PROs in early-phase oncology trials, lack of clarity on which PROs to select, and the absence of measure validation.	*"…I am concerned that the use of this data in a. small cohort of dose determination will risk undermining dose decisions."* (Clinician, 6-10 years experience)<br><br>*"…my experience with PRO-tools are that it takes 30minutes per questionnaire - it would not be feasible to collect this amount of data regularly during a defined DLT period; and without regular collection - I would not have confidence that the data is being recalled accurately."* (Clinician, 11-20 years experience)
	Lack of historical experience	Participants expressed concern over using PROs given the limited past examples of real-world use.	*"Lack of experience in this setting for using PRO is challenging to know how much weight to give PRO data."* (Regulator, 11-20 years experience)
	Subjectivity and lack of validation	PROs were considered inherently subjective and have not been validated against other established toxicity measures. Many participants cited this as a reason for their uncertainty about the utility of PROs in the dose-finding stage.	*"The evidence on the utility and the methodology of using PROs along with traditional methods is lacking."* (Statistician, >20 years experience)<br><br>*"They are not objective, as such any value function incorporating both CTCAE and PROs would likely be dominated by CTCAE."* (Statistician, 6-10 years experience)
	PRO selection	Regardless of sentiments towards PROs as a concept, 3 responses indicated a lack of guidance in moving from concept to selection and implementation.	*"Additionally with all the extra modules for the PRO tools, for a first in human study, it is not clear which modules to select (likely miss unexpected serious toxicity) "*

**Implementation issues**	Lack of training and resources	There is a lack of training and resources for implementing use and communicating between study teams.	*"[T]here is very rarely any mechanisms for the development teams to communicate with early phase 'research' teams."* (Trial manager/administrator)
	Data collection and monitoring	Participants expressed concerns over how the collection and monitoring of data could be done in a way that preserved data integrity.	*"…the [clinical research associates] often review these data not in real time, and raise lots of queries, which is impossible for the patient to recall, or the nurse/DM to resolve"* (Trial manager/administrator, 3-5 years experience)
	Lack of procedure	The lack of managerial support and established procedure for the PRO selection, data collection, data management, and communication within research teams make integration extremely difficult. This in turn leads to lost time and non-cohesive data.	*"The data cleaning took place so long after the patient was seen that neither doctor [nor] patient remembered exact details."* (Trial manager/administrator, 3-5 years experience)<br><br>*"…there is very rarely any mechanisms for the development teams to communicate with early phase 'research' teams."* (Trial manager/administrator, >20 years experience)
	Language accessibility	Individuals from non-English backgrounds will have additional trouble understanding and completing PROs. An effective tool that accommodates these participants will facilitate more diverse trial populations.	*"Will need to have PRO instruments that can cater to people who are not from English speaking backgrounds to ensure equity."* (Clinician, 0-2 years experience)
	Additional costs	The combination of additional training, process, and guidance captured in this theme also translate to additional costs to be incurred during conduct.	*"[S]tudy teams do not want yet another parameter holding up decision making progress given the financial / investor pressures."* (Trial manager/administrator, >20 years experience)
**Role of PROs**	Secondary measure	There was doubt about the value of PROs as a primary toxicity measure with respect to dose decisions, and many participants indicated that PROs may better serve as a secondary measure.	*"PROs should not be seen as the primary source of data for dose selection decision making. How these data and other key endpoints for dose selection decision making is not very well known."* (Statistician, 3-5 years experience)<br><br>*"It would be very challenging to integrate PROMs, other than as useful additional qualitative information, during escalation."* (Clinician, 6-10 years experience)
	Later phase trials	PROs may be best suited for expansion or later phase trials.	*"These are best used in expansion/later phase trials to collect more targeted safety information prior to registration trials"* (Clinician, 11-20 years experience)
	Case-by-case consideration	PROs should be used to make treatment decisions on a per-patient basis, rather than at the trial level.	*"…I am open to the per patient use of this data in determining ongoing care for the patient themselves."* (Clinician, 6-10 years experience)

	Dose refinement	Whether on a case-to-case basis or in an expansion phase, four participants agreed that PROs can be useful for dose refinement.	*"PROs have a huge role [in refining dose regimens]."* (Statistician, 11-20 years experience)<br>*"…my experience is people are more open to implement PRO at expansion or phase 2 rather than does escalation phase."* (Statistician, 6-10 years experience)
**Regulatory considerations**	Clinical value	The use of PROs data in making a regulatory decision would depend on the clinical significance.	*"If there is no anti-cancer activity the PRO data is not considered relevant."* (Regulator, 3-5 years experience)<br>*"…the "subjective" PRO data would never overthrow the [benefit/risk] balance as assessed by efficacy and safety, based on the "objective" investigator collection of adverse events, vital signs and laboratory results."* (Regulator, 11-20 years experience)
	Data reliability	The PROs data must have been collected reliably in order to be considered in decision-making.	*"…is the collection of the data likely to be sufficiently robust for decision making."* (Regulator, 11-20 years experience)
	Design-induced bias	Bias as a result of unblinding could affect interpretation of PRO data at the regulatory level.	*"Early phases are usually unblinded, making it difficult to ascertain objectivity."* (Regulator, 11-20 years experience)
	PRO selection	PROs need to have been appropriately selected to have meaning in regulatory decisions.	*"Depends on how well the PRO's have been thought through - are they appropriate / relevant etc"* (Regulator, 6-10 years experience)
